# Genome-Wide Identification of the *B*-*BOX* Genes that Respond to Multiple Ripening Related Signals in Sweet Cherry Fruit

**DOI:** 10.3390/ijms22041622

**Published:** 2021-02-05

**Authors:** Yanyan Wang, Zefeng Zhai, Yueting Sun, Chen Feng, Xiang Peng, Xiang Zhang, Yuqin Xiao, Xin Zhou, Weili Wang, Jiale Jiao, Tianhong Li

**Affiliations:** Department of Pomology, College of Horticulture, China Agricultural University, Beijing 100193, China; yabofei1212@163.com (Y.W.); zhaizefeng@126.com (Z.Z.); yuetingsun@126.com (Y.S.); fengc@cau.edu.cn (C.F.); rod@cau.edu.cn (X.P.); 18306391375@163.com (X.Z.); S20193172433@cau.edu.cn (Y.X.); zx51522zzwlwlbb@126.com (X.Z.); WWL0824@foxmail.com (W.W.); qingxuedanchen@163.com (J.J.)

**Keywords:** *Prunus avium*, *B-box*, fruit ripening, light, hormones

## Abstract

*B-BOX* proteins are zinc finger transcription factors that play important roles in plant growth, development, and abiotic stress responses. In this study, we identified 15 *PavBBX* genes in the genome database of sweet cherry. We systematically analyzed the gene structures, clustering characteristics, and expression patterns of these genes during fruit development and in response to light and various hormones. The *PavBBX* genes were divided into five subgroups. The promoter regions of the *PavBBX* genes contain *cis*-acting elements related to plant development, hormones, and stress. qRT-PCR revealed five upregulated and eight downregulated *PavBBX* genes during fruit development. In addition, *PavBBX6*, *PavBBX9*, and *PavBBX11* were upregulated in response to light induction. We also found that ABA, BR, and GA_3_ contents significantly increased in response to light induction. Furthermore, the expression of several *PavBBX* genes was highly correlated with the expression of anthocyanin biosynthesis genes, light-responsive genes, and genes that function in multiple hormone signaling pathways. Some *PavBBX* genes were strongly induced by ABA, GA, and BR treatment. Notably, *PavBBX6* and *PavBBX9* responded to all three hormones. Taken together, *BBX* proteins likely play major roles in regulating anthocyanin biosynthesis in sweet cherry fruit by integrating light, ABA, GA, and BR signaling pathways.

## 1. Introduction

Zinc finger proteins are transcription factors that play crucial roles in plants [1], including functions related to development and stress resistance [2]. *BBX* family zinc finger transcription factors have attracted increasing attention due to their multiple, important roles in plants [3,4]. For example, the *BBX* domains and CCT domains of *BBX* proteins play specific roles in transcriptional regulation and in protein interactions and nuclear transport, respectively [5,6]. Many studies have demonstrated that *BBX* proteins are essential for photomorphogenesis, floral induction, carotenoid biosynthesis, the shade avoidance response, and both biotic and abiotic stress resistance [3,7].

To date, 32 *BBX* proteins have been identified in *Arabidopsis thaliana*. These proteins were classified into five groups based on their domain characteristics [5] and are involved in regulating many biological processes, including flowering and photomorphogenesis [8,9,10]. CO/*AtBBX1* plays a central role in the photoperiodic regulation of flowering in Arabidopsis, and COL3/*AtBBX4* and COL9/*AtBBX7* participate in the regulation of flowering time [11,12]. Photomorphogenesis is positively regulated by *AtBBX11* [13], *AtBBX21* [3], *AtBBX22* [14], and *AtBBX23* [15] in Arabidopsis. By contrast, photomorphogenesis is negatively regulated by *AtBBX24* [16,17], *AtBBX25* [18], *AtBBX28/29* [19], *AtBBX30/31* [20]. In Arabidopsis. *HY5* is the core component of photomorphogenesis [21]. *AtBBX21* physically interact with *HY5*, and binds to the *T/G-box cis*-element in the promoter of *HY5* to activate its transcription [3]. Interesting, *HY5* can bind to the promoter region of *BBX11*, *BBX11* can also directly bind to the promoter region of *HY5* and positively regulate its expression. *BBX11-BBX21-HY5* form a positive feedback regulation system at the transcription level, which cooperatively promotes photomorphogenesis [13]. On the other hand, *HY5* binds to the promoters of *BBX30* and *BBX31* and inhibits their activity [22,23]. In addition, *BBX28* and *BBX29* negatively regulates photomorphogenesis by repressing the activity of transcription factor *HY5*, furthermore, both *BBX28* and *BBX29* interfere with the binding of *HY5* to the promoters of *BBX30* and *BBX31* [19,24,25]. *BBX28/BBX29*, *HY5*, and *BBX30/31* form a feedback loop to fine-tune photomorphogenic development [25]. Therefore, *BBXs* form a complex regulatory network with *HY5* to regulate photomorphogenesis [24].

*BBX* genes are also indispensable for abiotic stress responses and hormone signaling. *AtBBX24* is involved in salt stress signaling, as Arabidopsis plants overexpressing *AtBBX24* exhibited enhanced salt tolerance compared to wild-type plants [26]. In apple (*Malus domestica*), *MdBBX20* mediates UV-B and low temperature signaling to promote anthocyanin biosynthesis [27], and *MdBBX37* interacts with *MdMYB1* and *MdMYB9* to negatively regulate anthocyanin biosynthesis [28]. *PpBBX16* positively regulates light-induced anthocyanin accumulation in Asian pear (*Pyrus pyrifolia*) by activating *PpMYB10* expression [29]. *CmBBX19* interacts with ABF3 to negatively regulate drought tolerance in chrysanthemum (*Chrysanthemum morifolium*) [30]. In Arabidopsis, COL4/*AtBBX5* participates in salt stress responses through an ABA-dependent signaling pathway [31]. In addition, *AtBBX18* is involved in the gibberellic acid (GA) signal transduction pathway, and *AtBBX20* is involved in the brassinolide (BR) and light signal pathways [32,33]. *IbBBX24* improves the resistance of sweet potato (*Ipomoea batatas*) to Fusarium wilt via the jasmonic acid (JA) pathway [34].

Sweet cherry (*Prunus avium* L.), a fruit tree in the Rosaceae family, is one of the deciduous fruit trees [35]. Red-skinned sweet cherries are distinguished into dark-red and bicolored cultivars, and ‘Rainier’ is the one of the main bicolored cultivars recognized in the world, including in China. Our previous study demonstrated that light plays a major role in anthocyanin accumulation in bicolored but not dark-red cherries [36]. Therefore, in this study, ‘Rainier’ was used as the material to analyze the molecular mechanisms by which *BBX* genes participate in the regulation of light- and hormone-induced anthocyanin accumulation in sweet cherry, we identified and investigated 15 *PavBBX* family members in the genome database of sweet cherry. We analyzed their gene structures, phylogenetic relationships, and expression profiles in response to light and multiple hormone signals. Our results lay the foundation for further analyzing the roles of *BBX* genes sweet cherry fruit development and ripening.

## 2. Results

### 2.1. Identification of PavBBX Genes in Prunus avium

To identify the *BBX* genes in the sweet cherry genome, we used Arabidopsis *BBX* protein sequences as queries to search against the sweet cherry genome via BLAST. We verified the results of the BLAST search by confirming the presence of the *B-box* domain in each protein using SMART. After removing the redundant sequences, a total of 15 putative *PavBBX* genes were identified in sweet cherry, which were named *PavBBX1*-*PavBBX15* based on their locations in the reference genome (Figure S1). Detailed information about the *PavBBX* genes is provided in Table 1, including the gene name, protein length, chromosome location, molecular weight, theoretical isoelectric point, aliphatic index, and GRAVY value. The 15 *PavBBX* proteins had diverse molecular weights and lengths, ranging from 152 (*PavBBX6*) to 650 (*PavBBX1*) amino acids long. PavBBX6 had the lowest molecular weight (17.01 kDa), while *PavBBX1* had the highest (71.17 kDa). The theoretical isoelectric points of the *PavBBX* proteins ranged from 4.51 (*PavBBX8*) to 9.32 (*PavBBX4*), and the aliphatic indices ranged from 38.68 (*PavBBX8*) to 82.97 (*PavBBX15*). The GRAVY values of all *PavBBXs* were less than zero, pointing to the hydrophilic nature of *PavBBX* proteins (Table 1).

### 2.2. Protein Sequence Alignment and Phylogenetic Analysis of the PavBBX Gene Family

**Figure 1:**
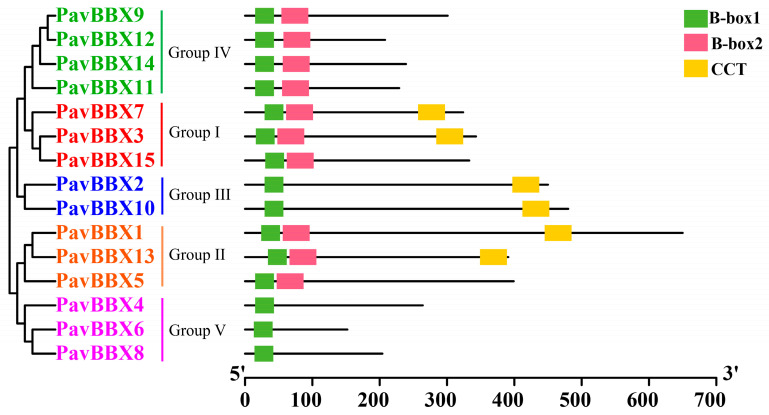
Structures of *Prunus avium BBX* proteins. The green, pink, and yellow rectangles represent the *B-box1*, *B-box2*, and CCT domains, respectively. The scale bar represents amino acids.

The lengths of the sweet cherry *BBX* proteins varied widely, from 152 to 650 amino acids. Among these, four *PavBBXs* contained two B-box domains and a conserved CCT domain. Overall, six *PavBBXs* contained two *B-box* domains but no CCT domain; additionally, two *PavBBXs* contained one B-box domain and a CCT domain, and three contained only one *B-box* domain (Figure 1). Protein sequence alignment and WebLogo analysis revealed that the CCT domains in the proteins were conserved and that the two *B-box* domains were highly homologous in different *BBX* proteins (Figures S2 and S3).

**Figure 2:**
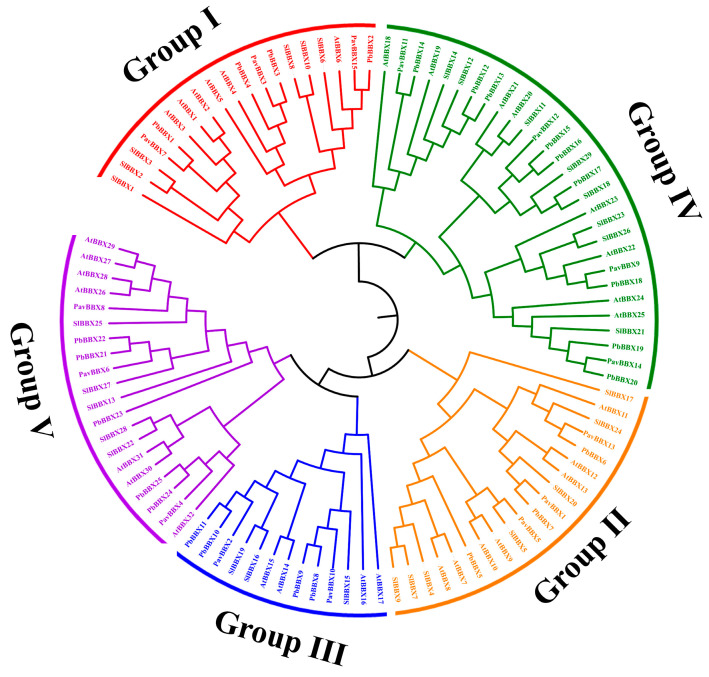
Phylogenetic analysis of *BBX* genes in sweet cherry (*Prunus avium*), pear (*Pyrus bretschneideri Rehd*), tomato (*Solanum lycopersicum*), and Arabidopsis (*Arabidopsis thaliana*).

To explore the evolutionary relationships and functional divergence of the *PavBBX* family members, we constructed a phylogenetic tree with MEGA6.0 using the neighbor-joining method based on various *PavBBX*, *AtBBX*, *PbBBX*, and *SlBBX* protein sequences (Figure 2). The *BBX* family was divided into five subgroups based on the results of phylogenetic analysis and previous studies in Arabidopsis [5], tomato [37] and pear [38]. *PavBBX* subgroup I and subgroup II members contain two *B-box* and one CCT domains, except for *PavBBX5* and *PavBBX15*. Subgroup III members contain one *B-box* and one CCT domain. *PavBBX* subgroup IV and V members contain two or one *B-box* domain (s) and no CCT domains.

### 2.3. Cis-Elements in the Promoters of Sweet Cherry BBX Genes

**Figure 3:**
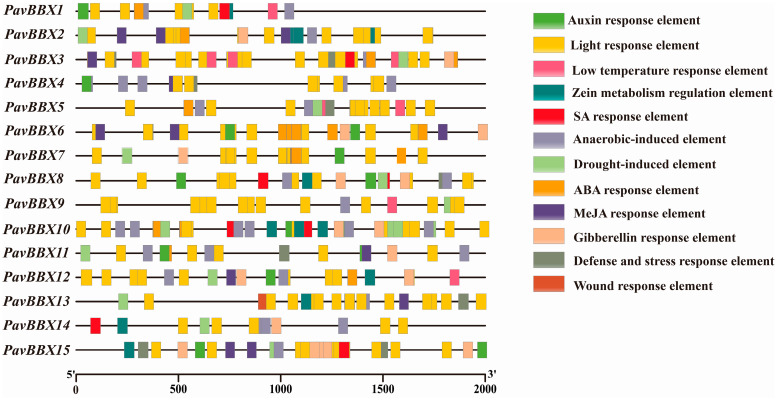
Schematic representation of the predicted regulatory *cis*-elements in the promoters of *PavBBX* family genes. The scale bar represents base pair.

*Cis*-elements are involved in regulating gene expression by interacting with their corresponding trans-regulators. Identifying proposed cis-elements would provide valuable information about the expression of the sweet cherry *BBX* genes. We therefore examined the promoter regions of the *PavBBX* genes and used the PlantCARE database for cis-element prediction (Figure 3). We identified 26 predicted cis-elements in these promoter regions. Among these, CAAT-box and TATA-box elements were present in all *PavBBX* genes. The remaining elements included light-responsive elements, defense response elements, and hormone-responsive elements, such as those induced by abscisic acid (ABA), auxin (IAA), gibberellin (GA), SA, and MeJA. These findings explained that *PavBBX* genes function in a variety of abiotic stress responses.

### 2.4. Expression Patterns of PavBBX Genes during Sweet Cherry Fruit Development and Ripening

**Figure 4:**
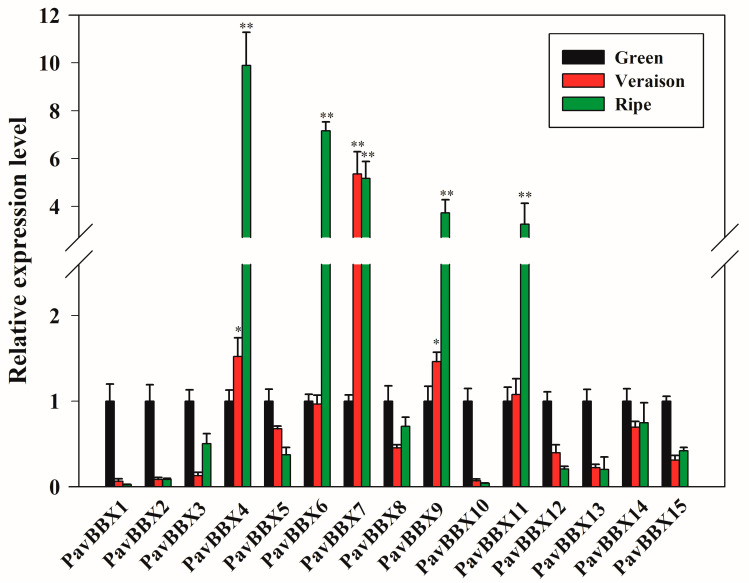
Expression profiles of the sweet cherry *PavBBX* genes during three stages of fruit development. *PavActin* was used as the internal reference control to normalize template levels. The relative mRNA levels are represented as the mean ± SD (n = 3). Statistically significant differences were assessed using Student’s *t*-test (* *p* < 0.05, ** *p* < 0.01).

To analyze the roles of *PavBBX* genes in fruit development and maturation, we examined the expression patterns of the 15 *PavBBX* genes in sweet cherry fruit during three developmental stages. Different *PavBBX* genes showed distinct expression patterns during different stages of fruit development. As shown in Figure 4, *PavBBX4*, *PavBBX6*, *PavBBX7*, *PavBBX9*, and *PavBBX11* were upregulated during fruit development, eight *PavBBX* genes were downregulated (*PavBBX1*, *2*, *3*, *5*, *10*, *12*, *13*, and *15*), and the expression of the two remaining genes did not significantly change during fruit development. These results speculated that some *PavBBX* genes play multiple, important roles in sweet cherry fruit development.

### 2.5. Regulation of PavBBX Gene Expression during Light Induction

**Figure 5:**
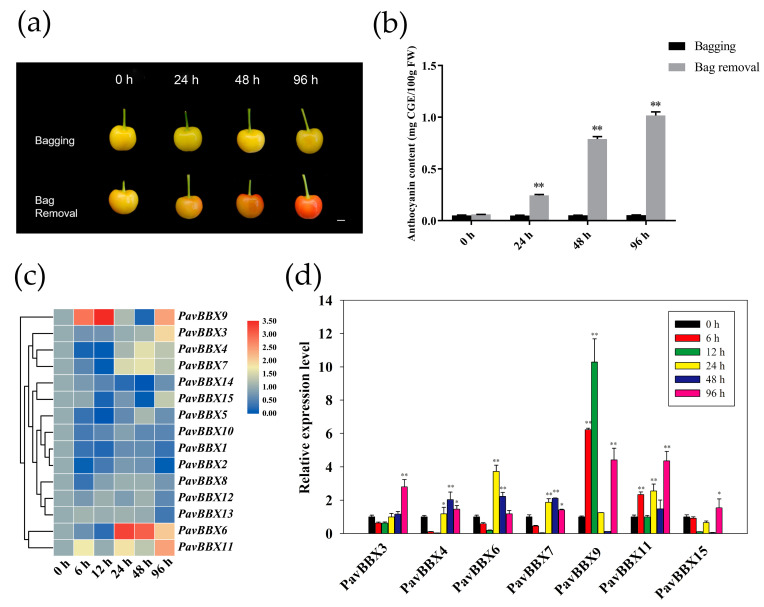
Expression profiles of *PavBBX* genes in ‘Rainier’ fruit following light induction via bag removal. (**a**) Color changes of ‘Rainier’ fruits after bag removal. Scale bars represent 1 cm. (**b**) Changes in anthocyanin content. (**c**) Hierarchical clustering of the expression profiles of 15 *PavBBX* genes after bag removing. (**d**) RT-qPCR analysis of seven selected *PavBBX* genes following light induction. *PavActin* was used as the internal reference control to normalize template levels. The relative mRNA levels are represented as the mean ± SD (n = 3). Statistically significant differences were assessed using Student’s *t*-test (* *p* < 0.05, ** *p* < 0.01).

*BBX* genes are involved in photomorphogenesis in a variety of plants [14,39]. Anthocyanin accumulation is highly dependent on light in bicolored Rainier cherries [36]. Therefore, to investigate the roles of the *PavBBX* genes in the plant response to light induction in bicolored ‘Rainier’ cherries, we placed bags on developing cherries at 15 DAF to block their exposure to light and removed the bags at 45 DAF. The fruits rapidly changed color after bag removal (Figure 5a), and anthocyanin continuously accumulated at 24 h after bag removal until the end of the experiment (96 h after bag removal) (Figure 5b). We used qRT-PCR to measure *PavBBX* genes expression in sweet cherry after bag removal. Different genes showed different expression levels (Figure 5c). As shown in Figure 5d, *PavBBX3*, *4*, *6*, *7*, *9*, *11*, and *15* were upregulated more than two-fold after light induction. In particular, *PavBBX9* was upregulated approximately 10-fold at 12 h of light induction and *PavBBX6* and *PavBBX11* were upregulated 4-5-fold at 24 h and 96 h of light induction, respectively. These results represented that these *BBX* genes function in light-induced anthocyanin biosynthesis in sweet cherry.

### 2.6. Light-Induced Regulation of Hormone Content in Sweet Cherry Fruit

**Figure 6:**
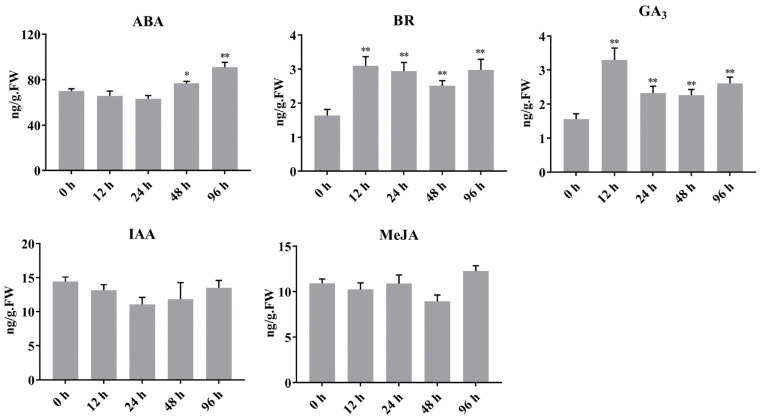
Changes in ABA, IAA, BR, MeJA, and GA_3_ contents in ‘Rainier’ fruits after bag removal. Error bars on each column represent the standard error (S.E.) of three replicates. Statistically significant differences were assessed using Student’s *t*-test (* *p* < 0.05, ** *p* < 0.01).

Plant hormones play important roles in inducing anthocyanin accumulation and commonly interact with light-signaling pathways to regulate plant growth [40]. Recent transcriptome expression studies have shown that anthocyanin biosynthesis in sweet cherry fruit involves plant hormone signaling, including ABA, auxin, BR, GA, and JA signaling pathways [36]. To determine whether light and plant hormones synergistically regulate anthocyanin accumulation in fruits, we measured hormone contents in ‘Rainier’ fruits after bag removal via ELISA. As shown in Figure 6, ABA contents significantly increased in fruits at 48 and 96 h after bag removal. In addition, BR and GA_3_ contents rapidly increased (2- to 3-fold) at 12 h after bag removal. However, IAA and MeJA contents did not significantly differ in fruits before and after bag removal. These results indicated that light regulates anthocyanin accumulation, at least in part, by modulating ABA, GA_3_, and BR signaling.

### 2.7. Co-Expression Network Analysis of PavBBX Genes with Anthocyanin Biosynthesis Genes, Light Signaling Genes, and Hormone Signaling Genes

**Figure 7:**
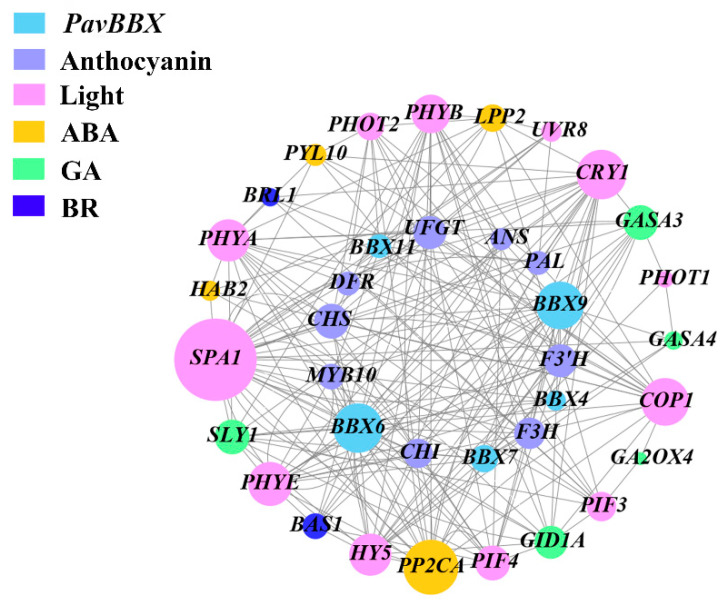
Co-expression network analysis of *BBX*, anthocyanin biosynthesis, light signaling, and multiple hormone signaling genes in bicolored cherry fruits.

We assessed the RNA-seq data (SAMN09296232 (RD50), SAMN09296233 (RL50) to examine the interactions between *BBX* genes, light-responsive genes, multiple hormone signaling genes, and anthocyanin biosynthesis genes in ‘Rainier’ fruits. The five *PavBBX* genes (*PavBBX4*, *6*, *7*, *9*, and *11*) were highly expressed during late fruit development and upregulated by light in ‘Rainier’ fruits. We analyzed the expression of nine anthocyanin biosynthesis genes (*PAL*, *CHS*, *CHI*, *F3H*, *F3′H*, *DFR*, *ANS*, *UFGT*, and *MYB10*) and 11 light-responsive genes to identify genes that might regulate anthocyanin biosynthesis during fruit development. As shown in Figure 7, genes encoding light signaling components (*HY5*, *PIF3*) and anthocyanin biosynthesis genes were co-expressed with ABA, GA, and BR signaling pathway genes, suggesting that these three hormones might play major roles in light-dependent anthocyanin biosynthesis in sweet cherry fruits. Furthermore, the expression patterns of five *PavBBX* genes (*PavBBX4*, *6*, *7*, *9*, and *11*) were correlated with the expression patterns of anthocyanin biosynthesis genes and light-responsive genes. Notably, *BBX6* and *BBX9* expression levels were highly correlated with anthocyanin biosynthesis and light-responsive gene expression, and these genes were co-expressed with genes encoding components of the ABA, BR, and GA signaling pathways. These results showed that *BBX* genes likely play major roles in regulating anthocyanin biosynthesis by integrating the light, ABA, GA, and BR signaling pathways.

### 2.8. Regulation of PavBBX Family Gene Expression during Hormone Treatment

**Figure 8:**
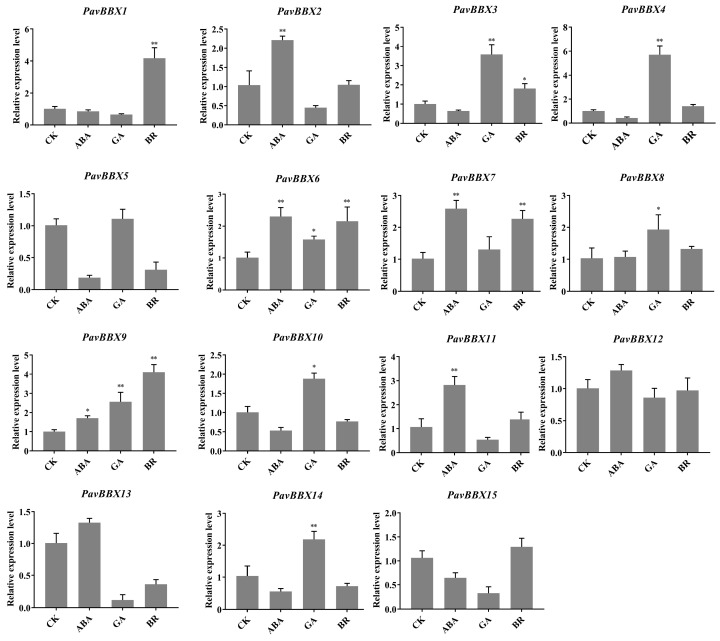
Expression profiles of sweet cherry *BBX* genes in response to ABA, GA, and BR treatment. *PavActin* was used as the internal reference control to normalize template levels. Relative mRNA levels are represented as the mean ± SD (n = 3). Statistically significant differences were assessed using Student’s *t*-test (* *p* < 0.05, ** *p* < 0.01).

Based on our analysis of changes in ABA, IAA, BR, MeJA, and GA_3_ contents in ‘Rainier’ fruits after light induction (Figure 6), we measured the expression levels of *PavBBX* genes in response to ABA, GA, and BR treatment by qRT-PCR (Figure 8). As expected, the *PavBBX* genes showed diverse expression patterns during treatment with different hormones. During ABA treatment, five *PavBBX* genes were significantly upregulated, indicating that these genes positively respond to ABA treatment. A majority of *PavBBX* genes responded to GA, including *PavBBX3*, *PavBBX4*, *PavBBX6*, *PavBBX8*, *PavBBX9*, *PavBBX10*, and *PavBBX14*, which were significantly upregulated under GA treatment. The 15 *PavBBX* genes responded to BR to varying degrees, including *PavBBX1* and *PavBBX9*, which were upregulated 4- to 5-fold compared to the control. Furthermore, *PavBBX6* and *PavBBX9* were significantly upregulated after treatment with the three hormones. In general, most *PavBBX* genes were sensitive to different hormone treatments.

### 2.9. Subcellular Localization of Sweet Cherry BBX Proteins

**Figure 9:**
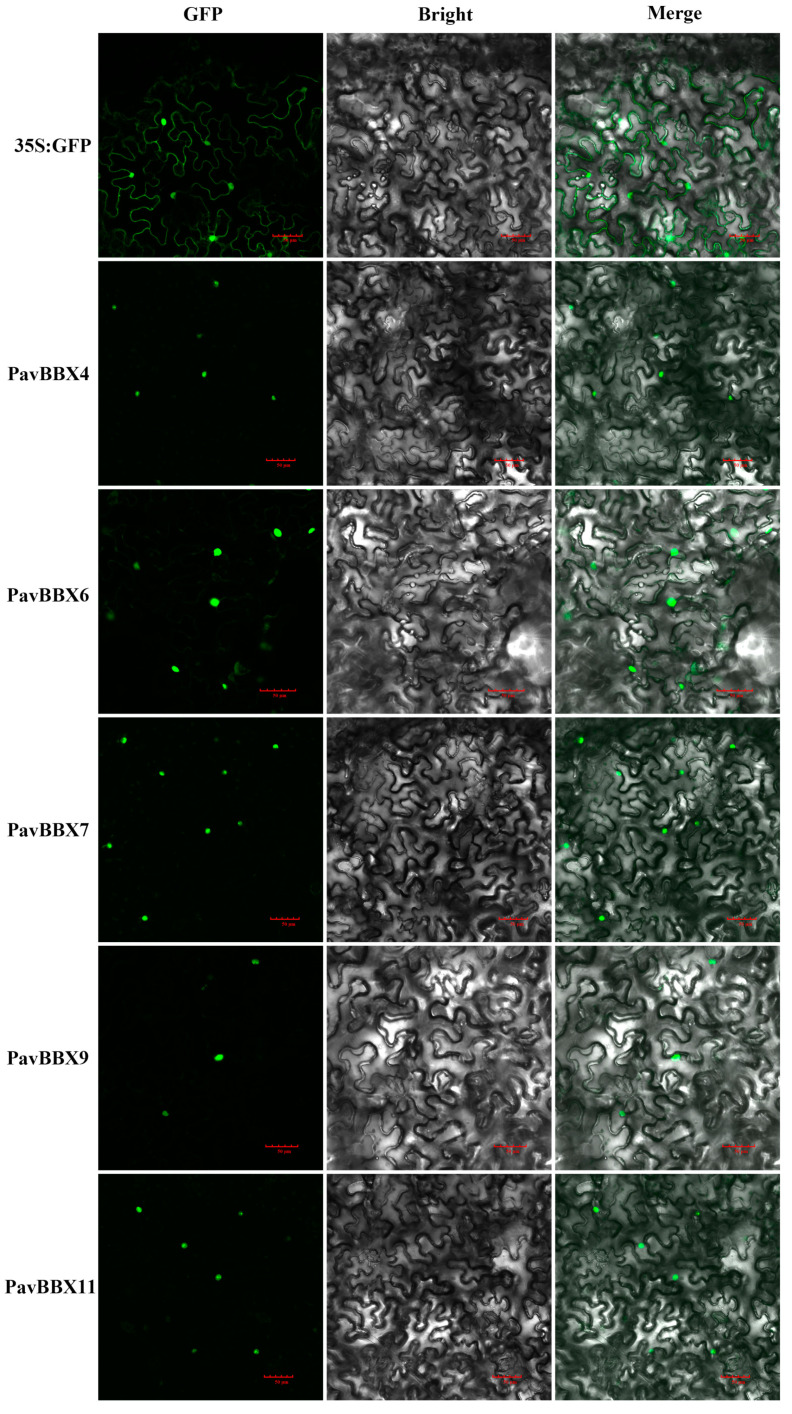
Subcellular localization of five GFP-fused *PavBBX* proteins. The five *PavBBX-GFP* fusion proteins (*PavBBX4-GFP*, *PavBBX6-GFP*, *PavBBX7-GFP*, *PavBBX9-GFP*, and *PavBBX11-GFP*) were transiently expressed in *N. benthamiana* leaves and observed by fluorescence microscopy 48 h later. Bar = 50 μm.

Transcription factors play many regulatory roles in plants. The nuclear localization of transcription factors is important for their regulatory roles. Most *BBX* proteins are located in the nucleus, such as *AtBBX21*, *AtBBX22* [3,14]. To examine the subcellular locations of *PavBBX* proteins in sweet cherry, we transiently transformed Nicotiana benthamiana epidermal cells with five *PavBBX* genes (*PavBBX4*, *6*, *7*, *9*, and *11*) and examined the subcellular localization of the resulting GFP-tagged fusion proteins. As shown in Figure 9, *PavBBX4-GFP*, *PavBBX6-GFP*, *PavBBX7-GFP*, *PavBBX9-GFP*, and *PavBBX11-GFP* showed green fluorescent signals in the nuclei of *N. benthamiana* epidermal cells. These results revealed that *PavBBX4*, *PavBBX6*, *PavBBX7*, *PavBBX9*, and *PavBBX11* are nuclear proteins, which is consistent with previous results and their presumed roles as transcription factors [3,14].

## 3. Discussion

### 3.1. Evolutionary Analysis of Sweet Cherry BBX Genes

In the current study, we identified 15 *BBX* genes in the genome database of sweet cherry. The number of *BBX* genes varies among plant species; for example, there are 32 *BBX* family members in Arabidopsis [5], 30 in rice [41], 64 in apple [42], 25 in pear [37], and 29 in tomato [38]. These differences may be due to the differences in genome size and complexity among these species. 

The *PavBBX* genes were divided into five subgroups based on multiple clustering analysis of *BBX* genes of different species [7]. Although the *BBX* genes in other species also fall into five subgroups, the number of genes in each subgroup differs among species. In *Arabidopsis thaliana*, 7, 8, 4, and 13 *AtBBX* genes contain one *B-box* domain, two *B-box* domains, one *B-box* domain and one CCT domain, and two *B-box* domains and one CCT domains, respectively, while the corresponding numbers in sweet cherry are 3, 6, 2, and 4, respectively. In addition, subgroup I and II members contain two *B-box* domains plus one CCT domains; subgroup III members contain one *B-box* domain and one CCT domain; subgroup IV members contain two *B-box* domains; and subgroup V members contain one *B-box* domain. It was difficult to classify subgroups in sweet cherry based on the presence of conserved domains (Figure 1 and Figure 2). *PavBBX5* and *PavBBX15* have no CCT domains even though they were assigned to subfamilies I and II; perhaps the CCT domain was lost from these genes during the process of evolution. The CCT domains are conserved and the two *B-box* domains are highly homologous in the different *BBX* genes (Figures S2 and S3), indicating that *BBX* genes arose early in the evolution of land plants.

### 3.2. The Expression Patterns of PavBBX Genes in Sweet Cherry Fruit Development and Ripening

Fruit development and ripening in sweet cherry is a complex physiological and biochemical process that is influenced by various transcription factors and regulatory proteins [36,43]. Many studies have shown that *BBX* genes are involved in anthocyanin biosynthesis and fruit ripening [14,28]. For example, *AtHY5* positively regulates anthocyanin biosynthesis [44], and *AtBBX22* interacts with *AtHY5* to promote anthocyanin biosynthesis [14]. The apple *B-box* protein *MdBBX37* modulates anthocyanin biosynthesis in conjunction with *MdMYB1*, *MdMYB9*, and *MdHY5* [28]. In grapevine, the expression of *VvBBX22* proposed its involvement in fruit development and hormone response [4]. By contrast, *MdBBX54* indirectly inhibits the expression of *MdMYB1* by interacting with *MdHY5* to suppress anthocyanin biosynthesis in apple [45]. In the current study, we demonstrated that *PavBBX9* (the homolog of *AtBBX22* in Arabidopsis) was significantly upregulated during late fruit development. This is consistent with previous findings [14]. Additionally, *PavBBX4*, *PavBBX6*, *PavBBX7*, and *PavBBX11* were upregulated during late fruit development, which revealed that these four genes are involved in the ripening process in sweet cherry. Finally, *PavBBX1*, *PavBBX2*, *PavBBX3*, and *PavBBX12* were downregulated during late fruit development, which means that these genes encode negative regulators of fruit ripening in sweet cherry.

### 3.3. The Expression Patterns of PavBBX Genes in Plant Responses to Light and Multiple Hormones

Light is an important factor that influences plant growth and development [40]. *BBX* proteins are primarily involved in seedling photomorphogenesis, the photoperiodic regulation of flowering, shade avoidance, and thermomorphogenesis [7,46,47,48]. These proteins also participate in the regulation of anthocyanin biosynthesis in fruits in the light [29]. For example, in pear, *PpBBX18* and *PpBBX21* antagonistically regulate light-induced anthocyanin biosynthesis via a competitive association with *PpHY5* [49], and *PpBBX16* regulates light-induced anthocyanin biosynthesis [29]. Additionally, in tomato, *SlBBX19*, *SlBBX20* and *SlBBX26* are light- and SlRIN-regulated, playing a role in fruit development ripening [50]. Anthocyanin accumulation is highly dependent on light in bicolored ‘Rainier’ cherries [36]. Based on our present results, seven *PavBBX* genes are upregulated during light exposure, proposed that these differentially expressed genes might function in light-induced anthocyanin biosynthesis.

Exogenous hormone treatment can promote fruit ripening [51]. Although sweet cherry has been classified as a non-climacteric fruit, ABA promotes the ripening of sweet cherry fruits, and ABA content in sweet cherry fruit increases strongly at the end of the color turning period and during the early stage of ripening [43]. The roles of *PavBBX* proteins in hormone signaling pathways are currently unclear. Several reports document the roles of *BBX* genes in hormonal pathways in other plants [39,52,53,54]. In Arabidopsis, *AtBBX21* regulates the light-mediated ABA signaling pathway by suppressing the transcriptional activation of *AtABI5*, leading to shorter hypocotyls [39]. *AtBBX24* might be involved in ethylene or brassinosteroid signaling based on its role in seedling photomorphogenesis in Arabidopsis [52]. Based on previous studies, we measured the hormone contents in ‘Rainier’ fruits that were bagged during fruit development, followed by bag removal, and found that ABA, GA_3_, and BR contents increased at 48 and 96 h after bag removal (Figure 6). Furthermore, the expression patterns of *BBX* genes were highly correlated with those of anthocyanin biosynthesis genes, light-responsive genes, and ABA, BR, and GA signaling pathway genes (Figure 7). RT-PCR revealed that the *PavBBX* genes were responsive to numerous hormonal treatments (Figure 8). The four *PavBBX* genes were regulated by more than one hormone treatment, providing that these genes may be involved in the interactions of different hormone signals at the physiological level. In addition, *PavBBX6* and *PavBBX9* responded to three hormone signals and light treatment, indicating that these genes encode proteins that integrate light and hormone signals to regulate anthocyanin biosynthesis. Together, these findings demonstrated that these *PavBBX* proteins might be involved in the crosstalk among multiple hormone signaling pathways and light, functioning as transcriptional regulators to modulate fruit development and ripening.

## 4. Materials and Methods

### 4.1. Plant Growth Conditions and Hormone Treatments

Bicolored ‘Rainier’ sweet cherry trees were grown under standard field conditions at the Beijing Institute of Forestry and Pomology, Beijing Academy of Agriculture and Forestry Sciences, Beijing, China. Fruit samples were collected at three time points during the growing season, including the green fruit expanding stage (15 days after flowering; DAF), veraison (40 DAF), and the ripe stage (50 DAF), and used to study the expression characteristics of *PavBBX* genes.

A fruit bagging experiment was conducted at 15 DAF. The bags were removed from sweet cherry fruits at 45 days after flowering (45 DAF). Samples were taken at 0, 6, 12, 24, 48, and 96 h after bag removal. There were three replicates performed in the experiment, with 10 fruits per replicate. The fruits were immediately frozen in liquid nitrogen and stored at −80 °C.

Young fruits at 36 DAF were surface sterilized with 75% alcohol for 1 min, rinsed twice with sterile distilled water, treated with 5% sodium hypochlorite for 13 min, and rinsed three times with sterile distilled water. The fruits were cut into small squares, placed on solid callus induction medium (MS medium + 2.0 mg/L 6BA, and 1.5 mg/L 2,4-D), and cultured for two weeks in the dark at 24 ± 2 °C. To investigate the effects of ABA, BR, and GA treatment, calli subcultured on standard medium for 15 days were treated with 100 μmol/L of each phytohormone and cultured in the light at 24 ± 2 °C under a 16 h/8 h light/dark cycle. After 5 days, all of the calli were immediately frozen in liquid nitrogen and stored at −80 °C for further analysis. All treatments were performed in three biological replicates.

### 4.2. Quantification of Total Anthocyanin Content

The total anthocyanin levels in cherries were measured as previously described [36]. The pigment was extracted with methanol containing 0.1% HCl in the dark at −20 °C overnight, centrifuged at 4 °C for 15 min at 8000 rpm, then the supernatant was obtained, and the content of total anthocyanins was determined by differential pH method, with the light absorbance of each sample measured at wavelengths of 510 and 700 nm using a UV–vis spectrophotometer (Shimadzu, Kyoto, Japan) in buffers at pH 1.0 and pH 4.5, respectively. Finally, the total anthocyanin content was expressed as mg cyanidin-3-o-rutinoside (CGE)/100 mg fresh weight (FW). All samples were performed in three replicates.

### 4.3. Identification of BBX Genes in the Prunus avium Genome

The deduced amino acid sequences of 32 *BBX* genes in *Arabidopsis thaliana* were used as query sequences for a BlastP search of the sweet cherry genome database (http://cherry.kazusa.or.jp/). The CDD database (https://www.ncbi.nlm.nih.gov/Structure/cdd/cdd.shtml) and the SMART database (http:// smart.embl-heidelberg.de) were used to analyze the domains of the candidate *BBX* proteins. The molecular weights, isoelectric points (pIs), and grand average of hydropathicity (GRAVY) values of the *PavBBX* proteins were calculated using the ExPASy website (https://web.expasy.org/protparam/).

### 4.4. Phylogenetic Analysis and Gene Structure Analysis

Multiple sequence alignments of BBX proteins were analyzed using ClustalW in BioEdit (http://bioedit.software.informer.com), and the phylogenetic tree was constructed with the neighbor-joining algorithm in MEGA 6.0 [55]. Bootstrap analysis was carried out with 1000 replicates. Domains were identified with the SMART (http://smart.embl-heidelberg.de) and Pfam (http://pfam.xfam.org) programs. WebLogo (http://weblogo.berkeley.edu/logo.cgi) was used to generate sequence logos of the conserved domains.

### 4.5. Cis-Element Prediction in the BBX Gene Promoters

The promoter sequences (2 kb upstream from ATG) were extracted from *Prunus avium* whole genome scaffolds data (version 1.0), and *cis*-elements in the promoters were predicted using the PlantCARE online program (http://bioinformatics.psb.ugent.be/webtools/plantcare/html/).

### 4.6. qRT-PCR Analysis

Total RNA was extracted from the samples using a Plant Total RNA Extraction kit (Huayueyang Biotechnology, Beijing, China). The integrity of the RNA was examined by 1.0% agarose gel electrophoresis, and cDNA was synthesized from the RNA using a Reverse Transcription kit (TaKaRa Biotechnology, Dalian, China). All primers used for qPCR are listed in Supplementary Table S1. qRT-PCR was performed using SYBR Premix Ex Taq (Kangwei Century Biotechnology, Beijing, China) with the Rotor-Gene Real-Time PCR System. RT-qPCR analyses were performed using the conditions recommended in MIQE guideline [56], we screened five potential reference genes. including *PavCAC*, *PavPP2A*, *PavSRP19*, *PavACT1,* and *PavActin* [36,57]. The detailed sequences are shown in Table S1, and we analyzed the variation between samples (*PavCAC*, <2.5 Cq, *PavPP2A* and *PavSRP19*, <2.0 Cq, *PavACT1*, and *PavActin* <1.5 Cq). *PavACT1* and *PavActin* were used presenting small variation between samples, and whose expression levels were expected to be constant over development and treatment. All experiments were carried out with three biological replicates. The relative expression value of each gene was quantified using the 2^–ΔΔCt^ method (relative to *PavActin*) [58].

### 4.7. Measuring ABA, IAA, MeJA, GA_3_, and BR Contents

The ABA, IAA, MeJA, GA_3_, and BR contents of the samples were measured using ELISA (enzyme-linked immunosorbent assay) as described by [59]. All measurements were performed in three biological replicates.

### 4.8. Co-Expression Network Construction and Visualization

The differentially expressed genes in the anthocyanin biosynthesis pathway, light signaling pathway, and hormone signaling-related pathways were identified in ‘Rainier’ sweet cherry (Table S2). Pearson’s correlation tests were performed with SPSS v25 software using the FPKM values of both SAMN09296232 (RD50) and SAMN09296233 (RL50) samples. Any two genes with an absolute Pearson correlation coefficient of ≥ 0.9 and a *p*-value of ≤0.05 were considered to be significantly co-expressed genes. The co-expression network was visualized using Cytoscape v3.5.1 software.

### 4.9. Subcellular Localization Analysis

To examine the subcellular locations of the *PavBBX* proteins, five full-length *PavBBX* open reading frames (ORFs) (for *PavBBX4*, *6*, *7*, *9*, and *11*) without the stop codon were amplified from cDNA from ‘Rainer’ fruit. Each amplification product was cloned into the pCAMBIA1302 vector with green fluorescence protein (GFP) label under the control of the *CaMV35S* promoter. The gene-specific primers are listed in Table S3.

*Nicotiana benthamiana* plants were grown in a plant growth chamber at 26 °C under a 16-h light/8-h dark regimen until they were approximately 15 cm tall and infiltrated with *Agrobacterium* strain EHA105 harboring the constructs described above. Infiltration was performed as described by [60]. The agroinfiltrated leaves were photographed 2 days after infiltration. GFP fluorescence images were captured using an Olympus laser-scanning confocal microscope.

## 5. Conclusions

In this study, we identified 15 *PavBBX* genes in the genome database of sweet cherry and systematically studied their gene structures and expression patterns. Our results illustrated that the *PavBBX* genes play important roles in fruit development and ripening, as revealed by *BBX* gene expression patterns and changes in hormone levels in the light, and especially by the correlation between the expression of several *BBX* genes and that of light-responsive genes, multiple hormones signaling genes, and anthocyanin biosynthesis genes. Notably, the expression levels of several *PavBBX* genes increased in response to various hormone treatments including ABA, GA, and BR, suggesting that these *BBX* genes regulate anthocyanin biosynthesis by integrating light, ABA, GA, and BR signaling pathways. Taken together, our genome-wide analysis of the *PavBBX* family lays the foundation for further research on the biological functions of these genes in fruit development and ripening.

## Figures and Tables

**Table 1:** *BBX* family genes in *Prunus avium*.

Gene Name	Accession Number	Protein/AA	Chrom	Chr Start	Chr End	MW (Da)	pI	Aliphatic Index	GRAVY
*PavBBX1*	Pav_sc0004527.1g080.1	650	Chr1	17737787	17743220	71,169.18	5.68	74.68	−0.458
*PavBBX2*	Pav_sc0001051.1g030.1	450	Chr1	24564167	24566102	49,926.26	5.15	56.84	−0.790
*PavBBX3*	Pav_sc0000131.1g850.1	343	Chr1	29989611	29991028	38,307.77	5.47	65.95	−0.539
*PavBBX4*	Pav_sc0000293.1g410.1	264	Chr3	661262	662056	28,357.08	9.32	72.16	−0.246
*PavBBX5*	Pav_sc0000405.1g160.1	399	Chr3	16146055	16148421	42,967.79	5.07	61.13	−0.485
*PavBBX6*	Pav_sc0001859.1g200.1	152	Chr3	16934620	16935833	17,011.06	9.09	60.46	−0.849
*PavBBX7*	Pav_sc0000051.1g110.1	324	Chr3	18112949	18115389	35,601.87	5.78	67.25	−0.482
*PavBBX8*	Pav_sc0000598.1g150.1	204	Chr4	1931059	1932064	23,047.58	4.51	38.68	−1.254
*PavBBX9*	Pav_sc0000352.1g070.1	301	Chr4	11752866	11755126	31,786.49	5.53	62.86	−0.317
*PavBBX10*	Pav_sc0000383.1g410.1	480	Chr5	15713586	15715861	53,565.68	5.12	62.23	−0.770
*PavBBX11*	Pav_sc0000044.1g810.1	229	Chr7	13871819	13874309	25,370.08	8.51	74.10	−0.387
*PavBBX12*	Pav_sc0001518.1g660.1	208	Chr8	8383526	8384850	23,011.66	5.97	58.17	−0.462
*PavBBX13*	Pav_sc0000848.1g470.1	391	Chr8	19897720	19900725	43,198.97	6.16	58.90	−0.661
*PavBBX14*	Pav_sc0001100.1g020.1	239	ChrUn	27735116	27739262	26,178.43	4.77	74.81	−0.315
*PavBBX15*	Pav_sc0002706.1g040.1	333	ChrUn	55031701	55033349	35,617.19	4.95	82.97	−0.046

## Data Availability

The Arabidopsis, pear and tomato *BBX* protein sequences were downloaded from the Arabidopsis information source (TAIR) database (http://www.arabidopsis.org), GigaDB database (http://gigadb.org/site/index) and Solanaceae genomics network (https://solgenomics.net/). The sweet cherry RNA-seq data in response to light were retrieved from NCBI database (SRA accession numbers: SRP149590 (https://www.ncbi.nlm.nih.gov/sra/SRP149590), respectively.

## Supplementary Materials

Supplementary materials can be found at https://www.mdpi.com/1422-0067/22/4/1622/s1. Figure S1: Chromosome distribution of sweet cherry *BBX* genes. The scale on the left is in megabases (Mb). Figure S2: WebLogos showing the conserved domains in PavBBX proteins. A, B, and C were obtained from protein sequence alignment of the *B-box 1*, *B-box 2*, and CCT domains, respectively. The x-axis indicates the conserved sequences of the domain. The height of each letter indicates the conservation of each residue across all proteins. The y-axis shows a scale of the relative entropy, which reflects the conservation rate of each amino acid. Figure S3: Multiple sequence alignments of the conserved domains of the *PavBBXs*. Multiple sequence alignments of the *B-box 1*, *B-box 2*, and CCT domains are shown. The sequences were aligned using DNAMAN 6.0 and BioEdit. Table S1: Primer sequences used for RT-qPCR. Table S2: Abbreviations of differentially expressed genes. Table S3: Primer sequences used for gene amplification.

## Abbreviations

*BBX*	*B-box*
ABA	Abscisic acid
MeJA	Methyl jasmonate
BR	Brassinolide
GA	Gibberellic acid
IAA	Indole-3-acetic acid
CCT	CO, CO-like, TOC1
DAF	Days after flowering
AA	amino acid residues
MW	molecular weight
pI	theoretical isoelectric point
GRAVY	grand average of hydropathicity
MS	Murashige and Skoog Medium
6BA	N-(Phenylmethyl)-9H-purin-6-amine
2,4-D	2,4-Dichlorophenoxyacetic acid
ELISA	Enzyme-linked immunosorbent assay
FPKM	Fragments per kilobase of exon per million mapped fragments
ORF	Open reading frame
GFP	Green fluorescent protein
MIQE	Minimum information stablished for qRT-PCR experiments
