# Supplementary figures and images for: Genome-Wide Identification of the B-BOX Genes that Respond to Multiple Ripening Related Signals in Sweet Cherry Fruit
Source: Int J Mol Sci. 2021 Feb 5;22(4):1622. doi: 10.3390/ijms22041622 (PMC7914455; doi:10.3390/ijms22041622)

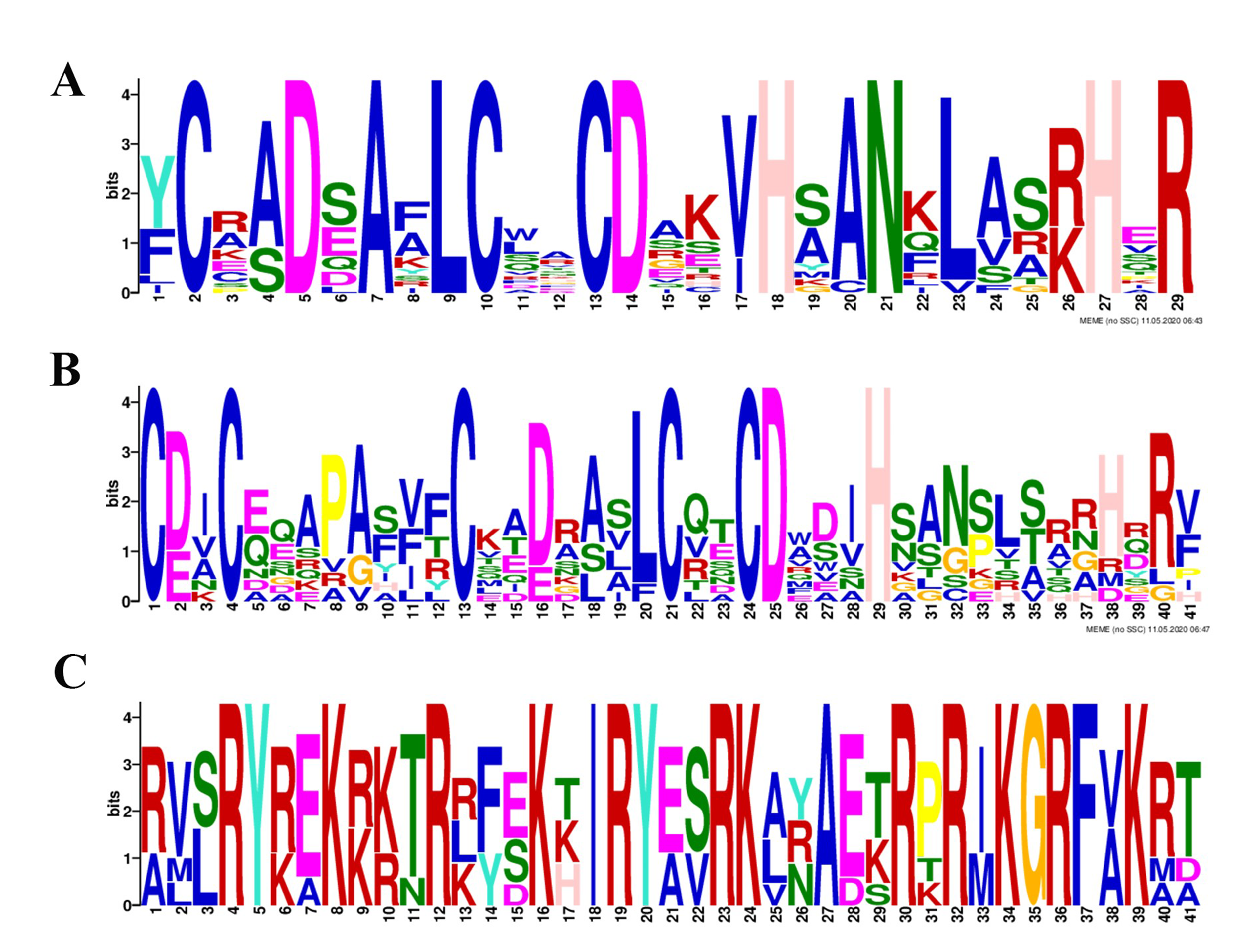

Supplement: Supplementary file 1 [file ijms-22-01622-s001.zip › Supplementary Information/Figure.S2.tif]

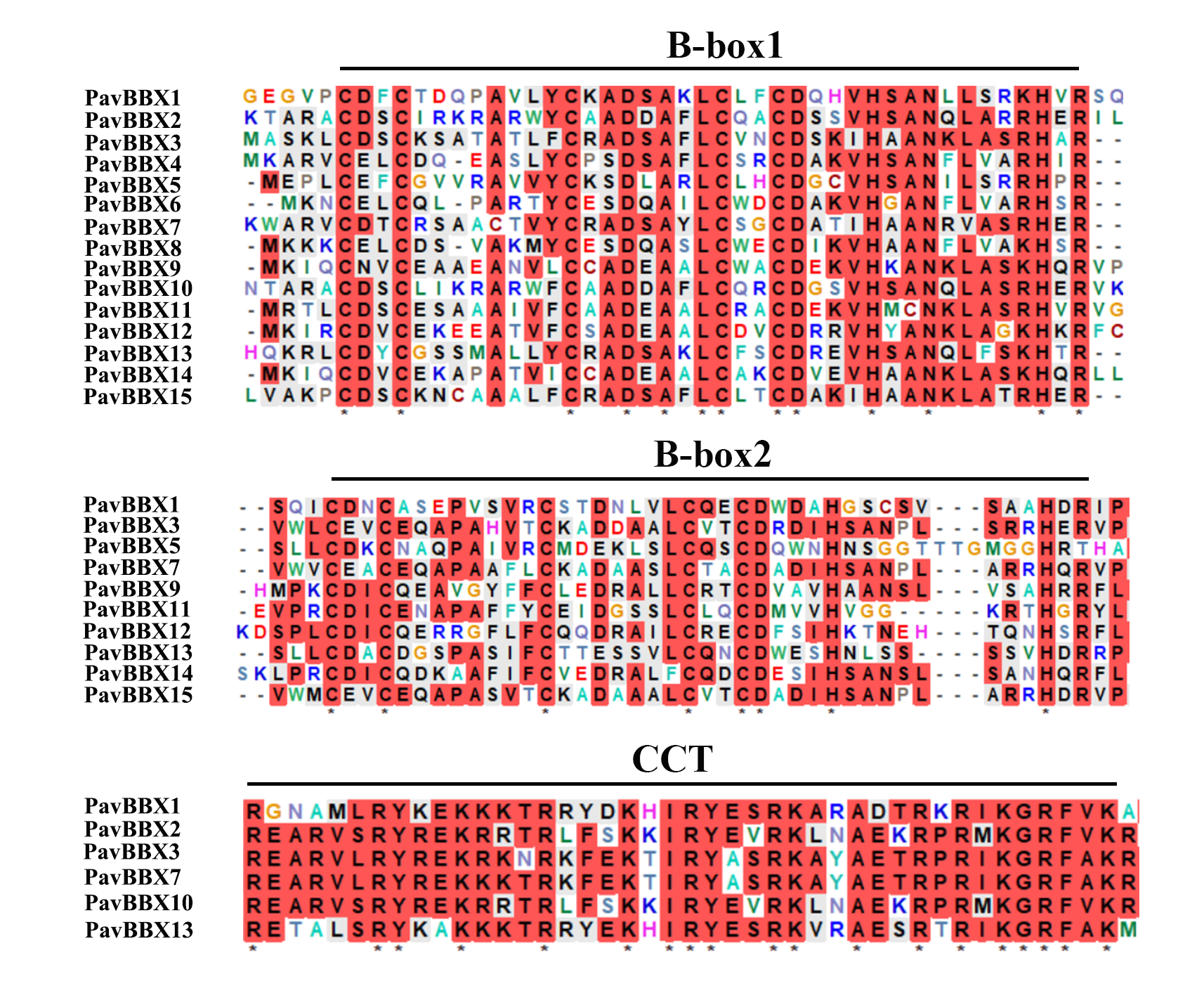

Supplement: Supplementary file 1 [file ijms-22-01622-s001.zip › Supplementary Information/Figure.S3.tif]
