# Supplementary material for: Genome-Wide Identification of the B-BOX Genes that Respond to Multiple Ripening Related Signals in Sweet Cherry Fruit
Source: Int J Mol Sci. 2021 Feb 5;22(4):1622. doi: 10.3390/ijms22041622 (PMC7914455; doi:10.3390/ijms22041622)
Supplement: Supplementary file 1 [file ijms-22-01622-s001.zip › Supplementary Information/Supplemental files.doc]

**Supplemental Figure**

**Figure.S1**: Chromosome distribution of sweet cherry *BBX* genes. The scale on the left is in megabases (Mb).

**Figure. S2**. WebLogos showing the conserved domains in PavBBX proteins. A, B, and C were obtained from protein sequence alignment of the B-box 1, B-box 2, and CCT domains, respectively. The x-axis indicates the conserved sequences of the domain. The height of each letter indicates the conservation of each residue across all proteins. The y-axis shows a scale of the relative entropy, which reflects the conservation rate of each amino acid.

**Figure. S3**. Multiple sequence alignments of the conserved domains of the PavBBXs. Multiple sequence alignments of the B-box 1, B-box 2, and CCT domains are shown. The sequences were aligned using DNAMAN6.0 and BioEdit.

**Supplemental Tables**

**Table S1. Primer sequences used for RT-qPCR**

| **Genes** | **Gene Accession** | **Forward Primer (5’to 3’)** | **Reverse Primer (5’to 3’)** |
| --- | --- | --- | --- |
| *PavBBX1* | Pav_sc0004527.1g080.1 | TACTTGCACAGAACAGGGGC | CCCAGCAAAACTCGACAACC |
| *PavBBX2* | Pav_sc0001051.1g030.1 | TCAACGACATGAGGAGGTGC | AGGCAAGCATTCGGAGTTCA |
| *PavBBX3* | Pav_sc0000131.1g850.1 | CCTTCCTCTGCGTCAACTGT | CTAAGAGGGTTGGCCGAGTG |
| *PavBBX4* | Pav_sc0000293.1g410.1 | CGTTGGGATTTTGCTTGGGG | AACGGCCAGGATCAGCTTAG |
| *PavBBX5* | Pav_sc0000405.1g160.1 | TGTAAATCGGACCTGGCTCG | TAATGCGTGGGTCCTATGCC |
| *PavBBX6* | Pav_sc0001859.1g200.1 | CCTCCGAAGGTCCCAAAACG | GTCCTGAGAGCGAAGATCCG |
| *PavBBX7* | Pav_sc0000051.1g110.1 | TGAGGCAGCATCATGGTTGT | GCTTCTGCATCTGAATCTGATTAT |
| *PavBBX8* | Pav_sc0000598.1g150.1 | TCAGCGTCACCTAAACAGAACA | CTCTTCCGGTCCTTGTGTCC |
| *PavBBX9* | Pav_sc0000352.1g070.1 | CTCTGTGCTGGGCTTGTGAT | GCAGCGTGTACAGCAACATC |
| *PavBBX10* | Pav_sc0000383.1g410.1 | GCAAGCAGACATGAGAGGGT | CCTTGCGTGTGAAACCCTTG |
| *PavBBX11* | Pav_sc0000044.1g810.1 | AGAGTGTTTTCAGTGTTCAGTGG | AAGCAGCAGATAACCGAGGC |
| *PavBBX12* | Pav_sc0001518.1g660.1 | GTGCGAGAAAGAAGAGGCCA | AACACGGCGATCACAGACAT |
| *PavBBX13* | Pav_sc0000848.1g470.1 | GTCATGGTCATGGAGGGCAA | TCCGCGATTCGTACCTGATG |
| *PavBBX14* | Pav_sc0001100.1g020.1 | CTGTGCGCCAAATGTGATGT | ACTCGGATTCCAGTGGCAAG |
| *PavBBX15* | Pav_sc0002706.1g040.1 | GCCTCCTCTATCGACTCCCT | CACTGTCCGTACCTGAACCC |
| *PavActin* | FJ560908 | CCAGGGCTGTGTTTCCTTCTA | ATGATCTGCGTCATCTTTTCT |
| *PavACT1* | XM_021976059 | CTCCTCTCAACCCTAAGGCTAACAG | CAGTTGTACGACCACTGGCATACAG |
| *PavCAC* | XM_021952533 | GCGTGCCAGACTGACCTT | GGCGAGCGTGACATATCTAACC |
| *PavPP2A* | XM_021963054 | CCCTCACAGCCCTTATTG | ATCGCACATTGGTCCTTC |
| *PavSRP19* | XM_021973246 | GGTTGTAATGGCTGGATTATATGG | GAAGGGAGACGAATAAGTGTAGAG |

**Table S2.** Abbreviations of differentially expressed genes

| **Type** | **Gene ID** | **Gene Name** | **Abbreviation** |
| --- | --- | --- | --- |
| Anthocyanin Biosynthesis | TR2081\|c0_g1 | Phenylalanine ammonia lyase | *PAL* |
|  | TR5133\|c0_g2 | Chalcone synthase | *CHS* |
|  | TR20120\|c0_g1 | Chalcone isomerase | *CHI* |
|  | TR3736\|c0_g1 | Flavanone 3-hydroxylase | *F3H* |
|  | TR4045\|c0_g1 | Flavanone 3’-hydroxylase | *F3'H* |
|  | TR9024\|c0_g1 | Dihydroflavonol-4-reductase | *DFR* |
|  | TR18948\|c0_g1 | Anthocyanidin synthase | *ANS* |
|  | TR9509\|c0_g1 | UDP-glucose: flavonoid-3-O-glucosyltransferase | *UFGT* |
|  | TR22565\|c0_g1 | MYB domain protein 10 | *MYB10* |
| Light Perception and Signaling | TR7872\|c0_g1 | Phytochrome A | *PHYA* |
|  | TR20444\|c0_g1 | Phytochrome B | *PHYB* |
|  | TR9262\|c0_g1 | Phytochrome E | *PHYE* |
|  | TR17268\|c0_g1 | Phytochrome E | *PHYE* |
|  | TR9262\|c1_g1 | Phytochrome E | *PHYE* |
|  | TR19584\|c0_g1 | Phytochrome E | *PHYE* |
|  | TR10801\|c0_g1 | Cryptochrome 1 | *CRY1* |
|  | TR24746\|c0_g2 | Cryptochrome 1 | *CRY1* |
|  | TR20780\|c0_g1 | Phototropin 1 | *PHOT1* |
|  | TR8691\|c0_g1 | Phototropin 2 | *PHOT2* |
|  | TR9000\|c0_g1 | UV resistance locus 8 | *UVR8* |
|  | TR13130\|c0_g1 | Constitutive photomorphogenic 1 | *COP1* |
|  | TR19947\|c0_g1 | Constitutive photomorphogenic 1 | *COP1* |
|  | TR19004\|c0_g1 | Elongated hypocotyl | *HY5* |
|  | TR20039\|c0_g1 | Phytochrome interacting factors 3 | *PIF3* |
|  | TR21747\|c0_g1 | Phytochrome interacting factors 3 | *PIF3* |
|  | TR10136\|c0_g1 | Phytochrome interacting factors 4 | *PIF4* |
|  | TR10136\|c1_g1 | phytochrome interacting factors 4 | *PIF4* |
|  | TR24838\|c0_g1 | Suppressor of PHYA 1 | *SPA1* |
|  | TR9819\|c0_g3 | Suppressor of PHYA 1 | *SPA1* |
|  | TR15200\|c0_g1 | Suppressor of PHYA 1 | *SPA1* |
| ABA Signaling | TR19389\|c0_g1 | Lipid phosphate phosphatase 2 | *LPP2* |
|  | TR11285\|c0_g1 | Arabidopsis PP2C-type phosphatase 1 | *AP2C1* |
|  | TR21235\|c2_g2 | Homology to ABI2 | *HAB2* |
|  | TR23334\|c0_g1 | Protein phosphatase 2CA | *PP2CA* |
|  | TR25434\|c0_g1 | Protein phosphatase 2CA | *PP2CA* |
|  | TR24123\|c0_g1 | PYR1-like 10 | *PYL10* |
| BR Signaling | TR19895\|c0_g1 | Brassinosteroid insensitive 1 | *BRL1* |
|  | TR8226\|c0_g3 | PHYB activation tagged suppressor 1 | *BAS1* |
| GA Signaling | TR12431\|c0_g1 | GA insensitive dwarf 1A | *GID1A* |
|  | TR777\|c0_g1 | Sleepy 1 | *SLY1* |
|  | TR23695\|c0_g2 | GAST1 protein homolog 3 | *GASA3* |
|  | TR19073\|c0_g1 | GAST1 protein homolog 3 | *GASA3* |
|  | TR23695\|c0_g1 | GAST1 protein homolog 4 | *GASA4* |
|  | TR22499\|c1_g1 | Gibberellin 2-oxidase 4 | *GA2OX4* |

**Table S3. Primer sequences used for gene amplification**

| **Genes** | **Forward Primer (5’to 3’)** | | | **Reverse Primer (5’to 3’)** | | |
| --- | --- | --- | --- | --- | --- | --- |
| *PavBBX4-*1302 | cacgggggactcttgaccatggtaATGAAAGCCAGGGTTTGC | | | ttctcctttactagtcagatctacACACTCGGCCCACCCCTC | | |
| *PavBBX6-*1302 | cacgggggactcttgaccatggtaATGAAGAACTGCGAGCTG | | | ttctcctttactagtcagatctacAGATCGAAGGCTATCT | | |
| *PavBBX7-*1302 | cacgggggactcttgaccatggtaATGTTGAAGGAAGAGAGC | | | ttctcctttactagtcagatctacGAATGATGGAACAATGCCAT | | |
| *PavBBX9-*1302 | cacgggggactcttgaccatggtaATGAAGATACAGTGCAAC | | | ttctcctttactagtcagatctacGAATTGCCTACGACGTTTT | | |
| *PavBBX11-*1302 | cacgggggactcttgaccatggtaATGCGAACACTTTGTGACTC | | | ttctcctttactagtcagatctacGTCCTCTTTATTTGAAGCTT | | |
|  | |  |  | |  |  |
